# Subcutaneous Emphysema and Postoperative Complications in Robot‐Assisted Gastrectomy: Impact of a Lower‐Pressure Insufflation Strategy

**DOI:** 10.1111/ases.70288

**Published:** 2026-04-13

**Authors:** Takashi Matsubara, Satoshi Takao, Shunsuke Kaji, Hiroki Okamura, Keisuke Inoue, Ayana Kishimoto, Kazunari Ishitobi, Takahito Taniura, Takayuki Tanaka, Tetsu Yamamoto, Masaaki Hidaka

**Affiliations:** ^1^ Department of Digestive and General Surgery Shimane University Izumo Japan

**Keywords:** pneumoperitoneum, robot‐assisted gastrectomy, subcutaneous emphysema

## Abstract

**Introduction:**

Subcutaneous emphysema (SE) after minimally invasive surgery is common, but its clinical significance in robot‐assisted gastrectomy remains unclear. We evaluated postoperative SE and explored the effect of a lower‐pressure insufflation strategy.

**Methods:**

We retrospectively reviewed 97 patients who underwent curative‐intent robot‐assisted gastrectomy for gastric cancer. SE on immediate postoperative abdominal radiographs was classified as negative or positive. Outcomes were compared between SE groups and between a standard‐pressure period (10 mmHg) and a lower‐pressure period (6–8 mmHg). Operative time was dichotomized using a 480‐min cutoff based on ROC analysis for CD grade ≥ II complications. AUCs were compared using the DeLong method. A supplementary analysis included the case number.

**Results:**

SE occurred in 37 of 97 patients (38.1%). CD grade ≥ II complications were more frequent in the SE‐positive than in the SE‐negative group (29.7% vs. 8.3%). In the primary parsimonious multivariable model, SE positivity and prolonged operative time were associated with CD grade ≥ II complications. Adding SE to operative time increased the AUC numerically, but not significantly (DeLong *p* = 0.52). Compared with the standard‐pressure period, the lower‐pressure period showed a lower SE incidence, fewer CD grade ≥ II complications, and a shorter operative time. In a supplementary analysis, prolonged operative time remained significant, whereas case number was not, and the association with SE was attenuated.

**Conclusion:**

Postoperative SE was common and may represent a marker of intraoperative physiologic or technical stress. A lower‐pressure insufflation strategy coincided with lower SE incidence and fewer clinically relevant complications. These findings are hypothesis‐generating.

## 1 Introduction

Robot‐assisted gastrectomy (RG) has been widely adopted as a minimally invasive approach that enables precise lymph node dissection and secure anastomosis through the use of multi‐jointed instruments and a three‐dimensional, high‐resolution view. However, the carbon dioxide (CO_2_) used to establish pneumoperitoneum can contribute to the development of subcutaneous emphysema (SE). SE is frequently observed on postoperative radiographs, but its clinical significance remains unclear [1, 2, 3, 4].

In urological, colorectal, and rectal cancer surgery, SE incidence rates of 10%–40% have been reported, with risk factors including advanced age, low body mass index (BMI), prolonged operative time, retroperitoneal manipulation, and extensive dissection [2, 3, 5]. Tamura et al. reported that advanced age and reduced subcutaneous fat thickness were associated with SE during robot‐assisted rectal resection [5], suggesting that decreased tissue resistance to gas infiltration is a common underlying mechanism.

In parallel, increasing societal demands to reduce CO_2_ emissions in healthcare have drawn attention to the environmental performance of pneumoperitoneum devices. The AirSeal system (CONMED, Utica, NY, USA) incorporates a valveless design and a three‐lumen access system and is characterized by (1) suppression of gas leakage, (2) stabilization of pneumoperitoneum pressure, (3) efficient smoke evacuation, and (4) reduced CO_2_ consumption [6]. In particular, lower‐pressure (6–8 mmHg) pneumoperitoneum pressure settings of 6–8 mmHg may decrease physiological stress and help suppress SE [7, 8, 9].

Nevertheless, the clinical impact of AirSeal remains controversial. Meta‐analyses by Lu et al. and Zhi et al., as well as randomized controlled trials by Desroches et al. and prospective trials and meta‐analyses in robot‐assisted partial nephrectomy, have suggested no or inconsistent differences in SE incidence or CO_2_‐related complications between AirSeal and conventional systems in urological and retroperitoneal procedures [8, 10, 11, 12, 13, 14, 15]. These findings imply that the effect of AirSeal may depend on the surgical procedure and patient characteristics.

Thus, while both the clinical relevance of SE and the benefits of AirSeal have been investigated in various fields, evidence specific to gastrectomy remains limited. Therefore, we conducted a retrospective study to determine whether the introduction of a lower‐pressure insufflation strategy reduced the incidence of postoperative SE and clinically relevant complications (Clavien–Dindo [CD] grade ≥ II) in robot‐assisted gastrectomy [16]. As a secondary analysis, we assessed the clinical significance of SE by comparing perioperative outcomes between SE‐positive and SE‐negative patients and explored factors associated with SE and CD grade ≥ II complications.

## 2 Patients and Methods

**FIGURE 1:**
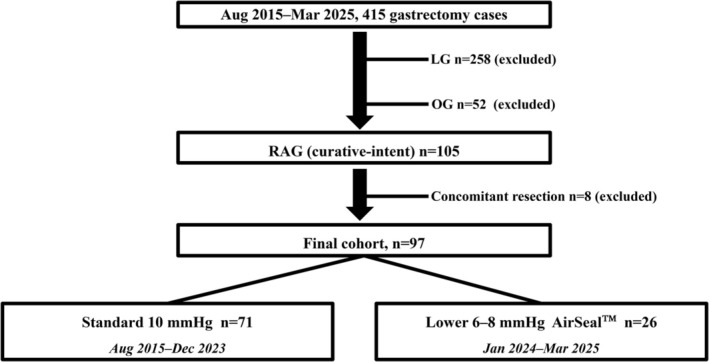
Patient selection flowchart. Between August 2015 and March 2025, 105 consecutive patients underwent curative‐intent robot‐assisted gastrectomy (RAG) at our institution. We excluded cases requiring concomitant organ resection other than cholecystectomy (*n* = 8), yielding a final analytical cohort of 97 patients. During the same period, laparoscopic gastrectomy (*n* = 258) and open gastrectomy (*n* = 52) were performed and were excluded from the present analysis because the study focused on RAG. For the period‐based analysis, included RAG cases were stratified into a standard‐pressure period (10 mmHg; August 2015–December 2023; *n* = 71) and a lower‐pressure period (6–8 mmHg with AirSeal; January 2024–March 2025; *n* = 26).

This single‐center retrospective study included 97 consecutive patients who underwent curative‐intent robot‐assisted gastrectomy (RAG) for histologically confirmed gastric adenocarcinoma between August 2015 and March 2025. During the same period, laparoscopic gastrectomy (*n* = 258) and open gastrectomy (*n* = 52) were performed but were not included in the analytical cohort. In total, 105 patients underwent curative‐intent RAG; cases requiring concomitant organ resection other than cholecystectomy were excluded (*n* = 8), yielding the final cohort of 97 patients. A patient selection flowchart is shown in Figure 1. There were no missing data for radiographic SE assessment or key postoperative outcomes.

All RAG procedures were performed using the da Vinci system by surgeons certified by the Japan Society for Endoscopic Surgery (JSES) endoscopic surgical skill qualification system (ESSQS) or deemed by the institution to have equivalent technical proficiency. The choice of surgical approach (robotic vs. laparoscopic vs. open) was not based on prespecified criteria or tumor stage and was determined by the attending surgeon. Surgeon assignment was based on institutional scheduling/credentialing and was not randomized.

Patients underwent routine preoperative oncologic staging (endoscopy with biopsy and contrast‐enhanced CT; additional tests such as EUS and/or PET‐CT when indicated) and standard physiologic assessment for surgical fitness. Perioperative management followed an institutional pathway (including nutritional/oral care support when indicated, early mobilization/respiratory therapy, prophylactic antibiotics, and standardized drain management), and postoperative surveillance was performed according to institutional practice.

**FIGURE 2:**
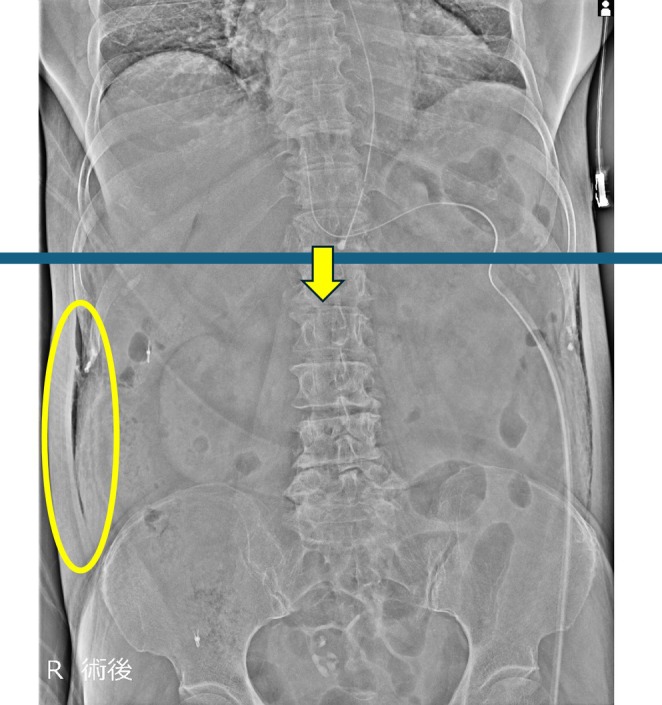
Representative postoperative abdominal radiograph of an SE‐negative case. Subcutaneous emphysema is confined to the abdominal wall at or below the level of the T12 thoracic vertebra (horizontal blue line). A small amount of gas is visible along the flank but does not extend cranially beyond T12.

**FIGURE 3:**
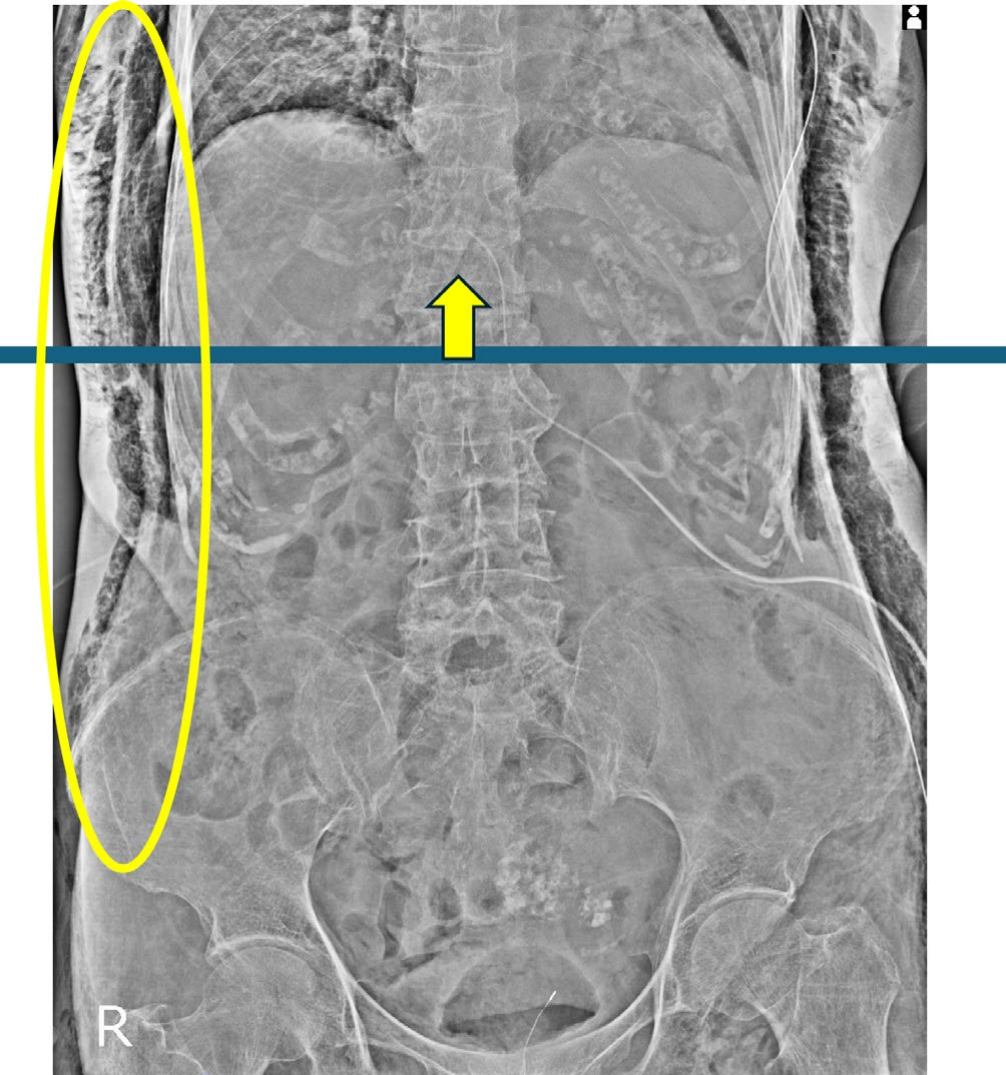
Representative postoperative abdominal radiograph of an SE‐positive case. Subcutaneous emphysema extends cranially beyond the level of T12, with gas tracking into the lower thoracic wall above the horizontal line. The arrows indicate the approximate level of T12 and the maximal cranial extent of subcutaneous emphysema.

Postoperative SE was assessed on the immediate postoperative supine abdominal radiograph obtained routinely after surgery (Figures 2 and 3). Because chest radiographs were not routinely obtained, thoracic/cervical SE may have been underestimated. Clinical variables (age, sex, BMI, procedure type, operative time) and postoperative complications (CD classification; ileus, organ/space SSI, respiratory complications, pancreatic fistula) were extracted from medical records. Given the limited number of CD grade ≥ II events (*n* = 16), we used a parsimonious multivariable logistic regression model to minimize overfitting; the primary model included only operative time and postoperative SE selected a priori.

In January 2024, we introduced a lower‐pressure insufflation strategy consisting of routine AirSeal use and a target pneumoperitoneum pressure of 6 mmHg, with temporary escalation to 8 mmHg permitted only when needed for safe manipulation such as minor hemostasis, followed by immediate return to 6 mmHg; no escalation beyond 8 mmHg. For the period‐based analysis, patients were stratified into the standard period (August 2015–December 2023; 10 mmHg, AirSeal not routinely used) and the lower‐pressure period (January 2024–March 2025; AirSeal used in all cases with the above protocol), and outcomes were compared between periods. Because AirSeal use was not consistently documented in historical standard‐period records, a sensitivity analysis restricted to AirSeal‐assisted procedures was not feasible.

Primary outcomes were the incidence of postoperative SE and CD grade ≥ II complications in the period‐based comparison. As a secondary (exploratory) analysis, outcomes were compared between SE‐positive and SE‐negative groups, and overall survival was evaluated using Kaplan–Meier estimates with the log‐rank test.

Statistical analyses were performed using JMP Pro software (SAS Institute, Cary, NC, USA). Continuous variables were compared between groups using the Mann–Whitney *U* test, and categorical variables were compared using the *χ* ^2^ test or Fisher's exact test as appropriate. Factors associated with SE and CD grade ≥ II postoperative complications were further evaluated using multivariable logistic regression analysis. A *p* < 0.05 was considered statistically significant. In the logistic regression analyses, operative time was entered as a dichotomized variable rather than as a continuous variable. The cut‐off value (480 min) was determined by receiver operating characteristic (ROC) curve analysis using CD grade ≥ II postoperative complications as the outcome. To evaluate whether the addition of postoperative SE improved discrimination beyond operative time alone, the areas under the two correlated ROC curves were formally compared using the DeLong method. In a supplementary multivariable logistic regression analysis, case number was included as an additional continuous covariate representing chronological case sequence.

### 2.1 Definitions

SE was assessed on the immediate postoperative supine abdominal radiograph. A horizontal reference line was defined at the level of the 12th thoracic vertebra (T12). SE‐positive was defined as subcutaneous gas tracking cranially beyond the T12 level, whereas SE‐negative was defined as the absence of subcutaneous gas or gas confined to the abdominal wall at or below T12. Clinically relevant postoperative complications were defined as CD grade ≥ II, that is, complications requiring pharmacologic treatment beyond routine care (e.g., antibiotics, blood transfusion, or total parenteral nutrition), endoscopic or interventional procedures, reoperation, or intensive care. SE was treated as a radiographic finding and was not counted as a postoperative complication unless it prompted additional therapeutic intervention.

### 2.2 Ethical Considerations

This study was conducted in accordance with the principles of the Declaration of Helsinki. The institutional review board approved the study protocol at our hospital, and the requirement for individual informed consent was waived owing to the study's retrospective design and the use of anonymized data.

## 3 Results

### 3.1 Patient Characteristics

Immediate postoperative supine abdominal radiographs revealed SE in 37 of 97 patients (38.1%). As shown in Table 1, patients in the SE‐positive group were significantly older than those in the SE‐negative group (median 74 vs. 72.5 years; *p* = 0.04). The proportion of female patients was also significantly higher in the SE‐positive group (45.9% vs. 21.7%; *p* = 0.01). There were no significant differences between the two groups in BMI (median 22.5 vs. 23.0 kg/m^2^; *p* = 0.12). However, operative time was significantly longer in the SE‐positive group than in the SE‐negative group (median 461 vs. 429.5 min; *p* = 0.03). Regarding the type of gastrectomy, the incidence of SE differed significantly among procedures (*p* < 0.05): SE occurred most frequently after total gastrectomy (54.5%) and least frequently after proximal gastrectomy (13.3%). Most cases of SE were confined to the abdominal wall, whereas in some patients, gas extended cranially beyond T12 on the abdominal radiograph. Mediastinal emphysema was rare and resolved with conservative management. No patient required invasive SE‐specific intervention.

**TABLE 1:** Patient characteristics and postoperative complications in SE‐negative versus SE‐positive groups undergoing robot‐assisted gastrectomy.

Category	SE negative (*N* = 60)	SE positive (*N* = 37)	*p*
Age	72.5	74	0.04
Sex			
M	47	20	0.01
F	13	17	
BMI	23.0	22.5	0.12
Operative time	429.5	461	0.03
Operative method			< 0.05
Distal	42	29	
Proximal	13	2	
Total	5	6	
Complication			
ALL grade			0.01
Negative	50	22	
Positive	10	15	
CD ≥ II			
Negative	55	26	0.01
Positive	5	11	
CD ≥ III			0.71
Negative	58	35	
Positive	2	2	
Pancreatic fistula			0.07
Negative	60	35	
Positive	0	2	
Respiratory complication			0.26
Negative	56	32	
Positive	4	5	
Postoperative ileus			0.37
Negative	56	36	
Positive	4	1	
SSI (organ)			0.02
Negative	60	34	
Positive	0	3	

### 3.2 Primary Analysis: Standard‐Pressure vs. Lower‐Pressure Periods

**FIGURE 4:**
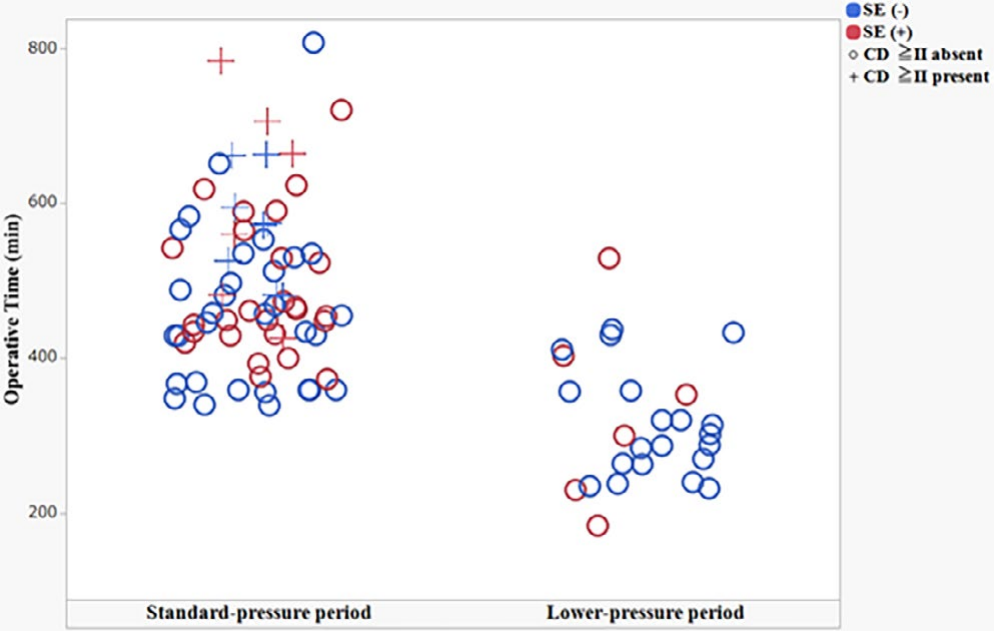
Operative time over chronological case order showing potential learning‐curve effects. Scatter plots show operative time plotted against chronological case order. Panels represent the standard‐pressure period (August 2015–December 2023) and the lower‐pressure period (January 2024–March 2025). Point color indicates postoperative subcutaneous emphysema (SE) status, and point shape indicates the presence of CD grade ≥ II complications.

A total of 71 patients underwent robotic gastrectomy during the standard‐pressure period and 26 during the lower‐pressure period (introduced at Case No. 67). In the lower‐pressure period, pneumoperitoneum at 6 mmHg was maintained throughout the procedure without escalation in 24 of 26 cases (92.3%). Temporary escalation to 8 mmHg was required in 2 of 26 cases (7.7%), both for hemostatic procedures for minor bleeding; no escalation was performed due to inadequate surgical exposure, and no case required escalation beyond 8 mmHg. Baseline characteristics were comparable between the two periods (Table 2). Median operative time decreased from 468 min in the standard‐pressure period to 301 min in the lower‐pressure period (*p* < 0.01). The incidence of SE decreased from 43.7% (31/71) to 23.1% (6/26) (*p* = 0.04). CD grade ≥ II complications occurred in 16 patients (22.5%) during the standard‐pressure period but in none during the lower‐pressure period (0/26; *p* = 0.01). Operative time showed a decreasing trend over chronological case order (Figure 4), and the distribution of SE and CD grade ≥ II complications across cases is also shown in Figure 4. Estimated blood loss did not differ significantly between the two periods.

**TABLE 2:** Comparison of perioperative outcomes between the standard‐pressure and lower‐pressure periods in robot‐assisted gastrectomy.

	Standard (10 mmHg)	Lower (6–8 mmHg)	*p*
Number of patients	71	26	
Age	74 [38–89]	71 [44–89]	0.7
Sex			0.15
Male	52	15	
Female	19	11	
BMI	22.8 [15.9–29.8]	22.8 [17.0–33.3]	0.54
ASA‐PS			0.2
≤ 2	65	24	
≥ 3	6	2	
Operative method			0.46
Distal	53	18	
Proximal	9	6	
Total	9	2	
Operative time	468 [339–807]	301 [184–529]	< 0.01
Estimated blood loss (mL)	2.5 [0–500]	0 [0–200]	0.12
SE			0.04
Positive	31	6	
Negative	40	20	
Complication (CD ≥ II)			0.01
Negative	55	26	
Positive	16	0	

### 3.3 Secondary Analysis: SE and Postoperative Complications

Postoperative complications of any grade were more frequent in the SE‐positive group than in the SE‐negative group (40.5% vs. 16.7%; *p* = 0.01). CD grade ≥ II complications were also more frequent in the SE‐positive group (29.7% vs. 8.3%; *p* = 0.01), whereas CD grade ≥ III complications did not differ significantly (5.4% vs. 3.3%; *p* = 0.71). Regarding specific complications, organ/space surgical site infection (SSI) occurred only in the SE‐positive group (3/37 vs. 0/60; *p* = 0.02). Pancreatic fistula tended to be more frequent in the SE‐positive group (2 vs. 0; *p* = 0.07). There were no significant differences in respiratory complications (5/37 vs. 4/60; *p* = 0.26) or postoperative ileus (1/37 vs. 4/60; *p* = 0.37). Among CD grade ≥ II complications (*n* = 16), respiratory complications were most frequent (*n* = 9), followed by postoperative ileus (*n* = 3), organ/space SSI (*n* = 2), and pancreatic fistula (*n* = 2). SE was treated as a radiographic finding and was not counted as a postoperative complication unless it prompted additional therapeutic intervention. In univariable logistic regression analysis, total gastrectomy, SE positivity, and prolonged operative time were associated with CD grade ≥ II complications. In a parsimonious multivariable model including only operative time and postoperative SE, both remained independently associated with CD grade ≥ II complications: operative time (OR = 23.4, 95% CI: 5.48–167.5; *p* < 0.001) and SE positivity (OR = 5.92, 95% CI: 1.62–25.1; *p* < 0.01) (Table 3). Model discrimination was numerically higher when postoperative SE was added to operative time (AUC 0.8792) than with operative time alone (AUC 0.8079); however, the difference between the two correlated ROC curves was not statistically significant by the DeLong test (*p* = 0.52).

**TABLE 3:** Univariable and multivariable analyses of risk factors for postoperative complications of Clavien–Dindo grade ≥ II.

Variable	Univariate		*p*	Multivariate		*p*
	OR	95% CI		OR	95% CI	
Age (> 75)	2.89	0.96–9.83	0.06			
Sex (female)	1.43	0.44–4.29	0.54			
cT (T2.3.4)	2.1	0.64–6.48	0.21			
cN (N1.2.3)	1.15	0.33–3.54	0.82			
Total gastrectomy	5.68	1.43–22.2	0.01			
SE (positive)	4.64	1.53–16.1	< 0.01	5.92	1.62–25.1	< 0.01
PNI (> 40)	1.79	0.25–8.72	0.51			
Operative time	20	5.05–134.3	< 0.01	23.4	5.48–167.5	< 0.01

### 3.4 Supplementary Analysis Including Case Number

In a supplementary multivariable logistic regression analysis including case number as a covariate representing chronological case sequence, prolonged operative time (> 480 min) remained significantly associated with CD grade ≥ II complications (OR = 33.11, 95% CI: 5.86–629.15; *p* < 0.0001), whereas case number was not significant (OR = 0.994 per case, 95% CI: 0.967–1.021; *p* = 0.6715). In contrast, the association with postoperative SE was attenuated and was no longer statistically significant (OR = 1.03, 95% CI: 0.27–3.82; *p* = 0.9696).

### 3.5 Exploratory Analysis: Overall Survival

**FIGURE 5:**
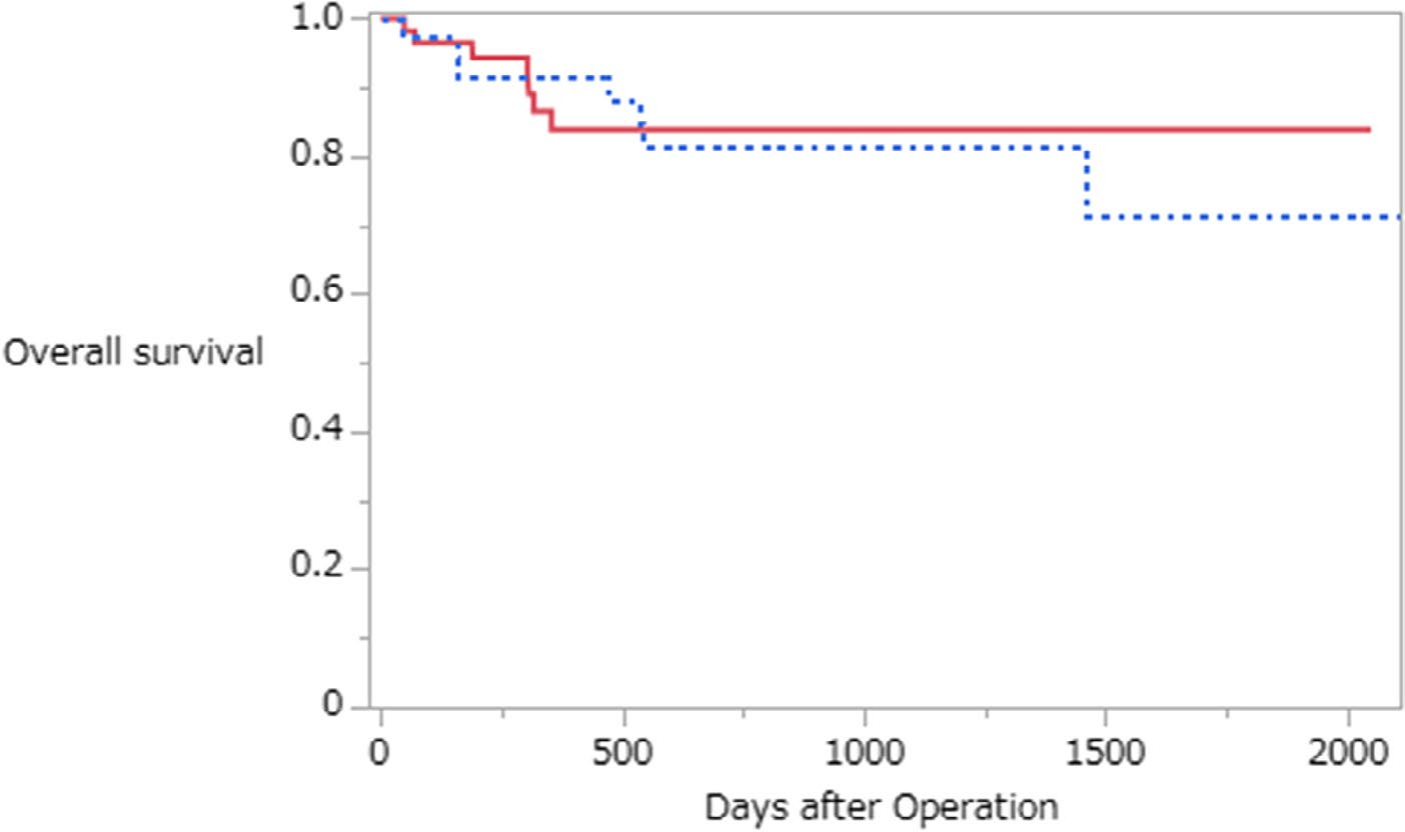
Kaplan–Meier curves of overall survival according to subcutaneous emphysema (SE) status. The solid line indicates SE‐negative, and the dotted line indicates SE‐positive. Overall survival (OS) was compared between SE‐positive and SE‐negative groups after robot‐assisted gastrectomy. There was no significant difference in OS between the two groups (log‐rank *p* = 0.71).

During a median follow‐up of approximately 2.5 years, Kaplan–Meier analysis showed no significant difference in overall survival between the SE‐positive and SE‐negative groups (log rank *p* = 0.71) (Figure 5).

## 4 Discussion

In this study, we clarified the incidence, risk factors, and association with postoperative complications of SE in robot‐assisted gastrectomy (RG) and evaluated the effect of introducing a lower‐pressure insufflation strategy (6–8 mmHg with routine AirSeal use). The incidence of SE was approximately 40% (37/97 patients), and advanced age, female sex, and total gastrectomy were significantly associated with SE occurrence. These factors are in line with reports from urological and laparoscopic surgery, in which vulnerability‐related factors have been shown to contribute to SE and related complications [2, 3, 5, 11, 12, 14]. Although SE is often regarded as a minor finding, it can lead to hypercapnia, mediastinal emphysema, pneumothorax, and respiratory depression, underscoring the need for careful clinical management [1, 2, 10, 17].

A central finding of the present study is that SE positivity was associated with an increased risk of CD grade ≥ II complications. However, SE should not necessarily be interpreted as a direct causal factor. Because operative time was associated with both SE and postoperative morbidity, operative time represents a major potential confounder that may also capture case complexity. SE may lie on the causal pathway linking unfavorable intraoperative conditions to postoperative complications. From this perspective, postoperative SE may be better interpreted as a clinically relevant marker or warning sign of intraoperative physiologic or technical stress rather than as an independent causal factor. To address this concern and avoid model overfitting, given the limited number of events (*n* = 16), we used a parsimonious multivariable model including only operative time and SE. In this primary model, SE positivity and prolonged operative time were associated with CD grade ≥ II complications. Model discrimination improved when SE was added to operative time alone (AUC 0.80787–0.87924); however, the difference was not statistically significant by the DeLong test (*p* = 0.52). Furthermore, in a supplementary analysis including case number as a covariate representing chronological case sequence, prolonged operative time (> 480 min) remained significantly associated with postoperative morbidity, whereas case number was not significant and the association with SE was attenuated and no longer statistically significant. These findings suggest that operative time may be the more robust predictor in this cohort, whereas SE may provide clinically relevant information as a marker of intraoperative physiologic stress or patient susceptibility not fully captured by operative time alone. Nevertheless, given the retrospective design and residual confounding by case complexity, these results should be interpreted as associations rather than definitive evidence of causality.

Clinically, organ/space SSIs and pancreatic fistulas were more frequent in the SE‐positive group, suggesting that CO_2_ tracking beyond the abdominal wall may contribute to tissue edema and local inflammatory changes. Prolonged maintenance of pneumoperitoneum has been reported to increase CO_2_ diffusion and absorption into tissues, resulting in elevated end‐tidal CO_2_ (EtCO_2_) and increased ventilatory burden [18]. The higher incidence of SE observed in this study during total gastrectomy is likely attributable to procedural factors, including frequent traction and suction maneuvers, expansion of the retroperitoneal space, and extensive dissection. In RG, liver retraction using a LAP PROTECTOR has been reported to confine SE to mild changes in the anterior chest and upper abdomen [19], suggesting that local preventive strategies tailored to the specific surgical technique are essential. Although respiratory complications were relatively frequent in this cohort, the present study does not establish a direct causal relationship between postoperative SE and these events. Rather, SE may reflect unfavorable intraoperative conditions, such as prolonged pneumoperitoneum exposure, gas dissection into tissue planes, hypercapnia‐related physiologic stress, or increased ventilatory burden, which could also predispose patients to postoperative respiratory morbidity.

Previous reports have documented SE‐related events such as mediastinal emphysema, pneumothorax, and CO_2_ narcosis [1, 10, 17], supporting the concept that SE should be regarded as a perioperative “warning sign” rather than a purely incidental radiographic finding. However, consistent conclusions regarding its relationship with severe morbidity (CD ≥ III) or long‐term prognosis remain limited. In thoracoscopic esophagectomy, postoperative SE has been linked to early complications and length of stay without a clear impact on long‐term survival [4], which aligns with our exploratory survival analysis. Taken together, SE may be best viewed as a marker of increased risk for moderate complications and delayed recovery rather than a determinant of fatal outcomes. During the lower‐pressure period, SE incidence decreased from 43.7% to 23.1%, and no CD grade ≥ II complications occurred. However, this period‐based comparison is inherently time‐dependent, and learning‐curve effects and temporal improvements in perioperative care likely contributed to reductions in operative time and postoperative morbidity. Therefore, between‐period differences should be interpreted cautiously and should not be construed as proof of an independent protective effect of lower‐pressure pneumoperitoneum. Importantly, the present comparison does not allow separation of the individual effects of pneumoperitoneum pressure and the insufflation device. The lower‐pressure period was also characterized by routine AirSeal use, whereas AirSeal use during the standard‐pressure period was inconsistent and not systematically documented. Therefore, the observed differences should be interpreted as reflecting the effect of a combined insufflation strategy rather than the isolated effect of either factor alone. The finding that 6 mmHg pneumoperitoneum was maintained in 92% of cases is encouraging but should be interpreted cautiously. This high feasibility may have been influenced by case selection, accumulated team experience, and learning‐curve effects during the later study period and therefore may not be directly generalizable to all institutions or surgical teams. Nevertheless, within our robotic gastrectomy setting, these data suggest that routine insufflation pressures ≥ 8 mmHg may not be necessary in many cases, as 6 mmHg was sufficient in the majority of patients with only temporary escalation when required. Our findings are best viewed as hypothesis‐generating and supportive of further evaluation of a lower‐pressure insufflation strategy in RG.

Our results raise the possibility that a lower‐pressure insufflation strategy may be particularly beneficial in patients at high risk of SE. Reducing CO_2_ emissions in the operating room has attracted increasing international attention as part of “Green Theater” initiatives. Given its minimal gas leakage, AirSeal may reduce CO_2_ consumption, and several clinical and experimental studies have demonstrated reduced CO_2_ absorption and diffusion during laparoscopy and prolonged pneumoperitoneum when using valveless systems [18, 20]. Sustainability guidelines from the Royal College of Surgeons and the European Association for Endoscopic Surgery (EAES) also recommend low‐pressure pneumoperitoneum, reduced gas usage, and efficient smoke evacuation systems [21, 22]. Thus, a lower‐pressure insufflation strategy may have the potential to promote both patient safety and environmental sustainability. However, total CO_2_ usage was not directly measured in this study, and a quantitative assessment will be essential in future evaluations of ecological burden.

This study has several limitations. First, it is a single‐center retrospective analysis with a limited number of events. Although we performed a supplementary analysis including case number as a covariate representing chronological case sequence, residual time‐trend confounding cannot be fully excluded. Second, SE was assessed using immediate postoperative abdominal radiographs and dichotomized using a pragmatic T12‐based definition; thoracic/cervical SE may have been underestimated, and SE severity was not quantified. Third, surgeon allocation was not randomized and was influenced by scheduling, credentialing, and perceived case complexity. Fourth, because AirSeal use in the standard period was not consistently traceable, a sensitivity analysis restricted to AirSeal‐assisted cases could not be performed. Multicenter prospective studies incorporating standardized SE quantification and detailed intraoperative physiologic and gas‐consumption data are warranted to clarify mechanisms and validate the clinical and environmental implications of lower‐pressure insufflation strategies.

## 5 Conclusions

In this retrospective study, postoperative SE was common after robot‐assisted gastrectomy and may represent a marker of intraoperative physiologic or technical stress. A lower‐pressure insufflation strategy was observed together with lower SE incidence, shorter operative time, and fewer clinically relevant postoperative complications. However, supplementary analysis suggested that prolonged operative time was the more robust predictor of postoperative morbidity. Therefore, the present findings should be interpreted as hypothesis‐generating and warrant further prospective validation.

## Funding

The authors have nothing to report.

## Conflicts of Interest

The authors declare no conflicts of interest.

## Data Availability

The data that support the findings of this study are available on request from the corresponding author. The data are not publicly available due to privacy or ethical restrictions.
